# S100A12 in Digestive Diseases and Health: A Scoping Review

**DOI:** 10.1155/2020/2868373

**Published:** 2020-02-26

**Authors:** Alexandre Carvalho, Jacky Lu, Jamisha D. Francis, Rebecca E. Moore, Kathryn P. Haley, Ryan S. Doster, Steven D. Townsend, Jeremiah G. Johnson, Steven M. Damo, Jennifer A. Gaddy

**Affiliations:** ^1^Internal Medicine Program, St. Joseph Mercy Hospital, Ann Arbor, Michigan, USA; ^2^Department of Pathology, Microbiology, And Immunology, Vanderbilt University School of Medicine, Nashville, Tennessee, USA; ^3^Department of Chemistry, Vanderbilt University, Nashville, Tennessee, USA; ^4^Department of Biomedical Sciences, Grand Valley State University, Allendale, Michigan, USA; ^5^Division of Infectious Diseases, Department of Medicine, Vanderbilt University Medical Center, Nashville, Tennessee, USA; ^6^Department of Microbiology, University of Tennessee, Knoxville, Tennessee, USA; ^7^Department of Life and Physical Sciences, Fisk University, Nashville, Tennessee, USA; ^8^Departments of Biochemistry and Chemistry, Vanderbilt University, Nashville, Tennessee, USA; ^9^Department of Structural Biology, Vanderbilt University, Nashville, Tennessee, USA; ^10^Tennessee Valley Healthcare Systems, Department of Veterans Affairs, Nashville, Tennessee, USA

## Abstract

Calgranulin proteins are an important class of molecules involved in innate immunity. These members of the S100 class of the EF-hand family of calcium-binding proteins have numerous cellular and antimicrobial functions. One protein in particular, S100A12 (also called EN-RAGE or calgranulin C), is highly abundant in neutrophils during acute inflammation and has been implicated in immune regulation. Structure-function analyses reveal that S100A12 has the capacity to bind calcium, zinc, and copper, processes that contribute to nutritional immunity against invading microbial pathogens. S100A12 is a ligand for the receptor for advanced glycation end products (RAGE), toll-like receptor 4 (TLR4), and CD36, which promote cellular and immunological pathways to alter inflammation. We conducted a scoping review of the existing literature to define what is known about the association of S100A12 with digestive disease and health. Results suggest that S100A12 is implicated in gastroenteritis, necrotizing enterocolitis, gastritis, gastric cancer, Crohn's disease, irritable bowel syndrome, inflammatory bowel disease, and digestive tract cancers. Together, these results reveal S100A12 is an important molecule broadly associated with the pathogenesis of digestive diseases.

## 1. Introduction

The human protein S100A12 (also named calgranulin C and EN-RAGE) is primarily expressed and secreted by granulocytes such as neutrophils [1, 2]. S100A12 belongs to the S100 family of EF-hand calcium-binding proteins, which participate in a wide variety of intracellular and extracellular functions. There are over two dozen identified S100 family proteins, and several have the capacity to form dimers, participate in Ca^2+^ signaling, and regulate numerous cellular processes including calcium homeostasis, energy metabolism, and cell proliferation and differentiation [1, 2]. Some S100 family proteins act as damage-associated molecular patterns (DAMPs), which are molecules released by stressed cells undergoing necrosis which act as endogenous signals to promote a proinflammatory response, and interact with pattern recognition receptors (PRRs) to modulate cellular responses [3–11]. Additionally, some S100 proteins have potent immunoregulatory and antimicrobial functions and induce signal transduction and cell proliferation, making them critical components of the innate immune system [3–8]. Specifically, S100A12 has been shown to initiate proinflammatory and antimicrobial responses in the gastrointestinal tract. In this scoping review, we provide insight into structure, function, and associations of S100A12 with gastrointestinal health and disease.

### 1.1. S100A12 Association with Granulocytes

The S100A12 protein was first discovered as a calcium-binding protein isolated from porcine granulocytes [12]. S100A12 is coexpressed with two other S100 proteins, S100A8 (Mrp8) and S100A9 (Mrp14), the two subunits of the calprotectin heterodimer, within granulocytes [8]. S100A12 and S100A8/A9 proteins are encoded on the same chromosome, appear to be coregulated, and have functional and structural similarities [8, 13]. Additionally, recent studies have revealed that S100A12 interacts with S100A9 [14]. S100A12 is expressed in neutrophils, macrophages, and lymphocytes and is secreted by neutrophils as an innate immune response against microorganisms and parasites [7, 15]. S100A12 is constitutively expressed in neutrophils but is inducible in other cell types including epithelial cells [16]. S100A12 lacks signal peptides required for the canonical Golgi-mediated secretion pathway, but secretion of S100A12 from neutrophils instead involves reactive oxygen species (ROS) and potassium (K(+)) exchanges through the ATP-sensitive K(+) channel [8].

### 1.2. S100A12 Structure

**Figure 1:**
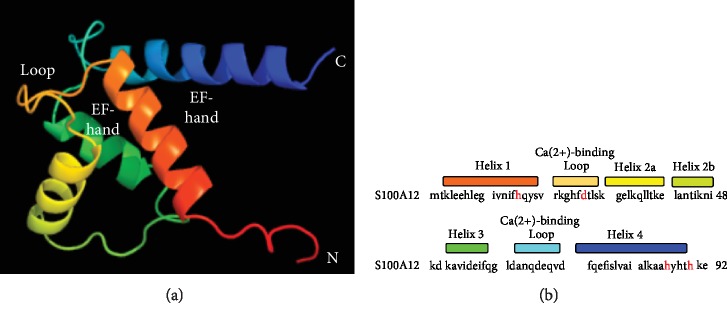
Model of S100A12 structure. (a) indicates PHYRE prediction of S100A12 secondary and tertiary structure. (b) indicates the primary structure beginning with helix 1 at the N terminus (pictured above in orange), calcium-binding loop 1 (pictured in yellow-orange), helix 2 (pictured in yellow), helix 3 (pictured in green), calcium-binding loop 2 (pictured in turquoise), and helix 4 (pictured in blue). The metal-binding residues which comprise the dimer interface are highlighted in red in the primary structure sequence. The two EF-hand motifs, similar in structure to a thumb and forefinger, are pictured in orange/yellow and green/blue, respectively. Metal-binding residues are highlighted in red.

Analysis of crystal structures reveals that S100A12 monomeric subunits have four *α*-helices in a H1–H2–H2′–H3–H4 topology and two EF-hand motifs (EF-1 and EF-2) connected by loop L2, the so-called “hinge” region (see Figure 1). In the presence of calcium, these form an “open” conformation between helices H3 and H4 [17, 18]. Furthermore, the C-terminal loop (His^88^-Glu^92^), which adopts an orientation in contact with the hinge region and helix H4, blocks the active sites in S100 proteins, at calcium concentrations of 5 *μ*M. Interestingly, the crystal structures of Zn^2+^ and Ca^2+^/Cu^2+^-bound S100A12 indicate that zinc and copper share the same binding site on human S100A12, and metal binding has the capacity to shift the C-terminal loops of both apo- and Ca^2+^-loaded S100A12 and extend the length of helix H4 from His^88^ to His^90^ [19, 20]. These data suggest that zinc/copper interactions lead to the Ca^2+^-loaded S100A12 adopting a “completely open” structure with larger interhelical angles between helices H3 and H4 for increased target recognition [21]. Crystal structures also reveal that S100A12 exists in a dimer or hexamer form in the presence of 200 *μ*M CaCl_2_ and that the addition of zinc can induce hexamerization [22–26]. Also, S100A12 structural stability is increased with the addition of Zn^2+^ and Ca^2+^ [22–24].

### 1.3. The Functions of S100A12

**Figure 2:**
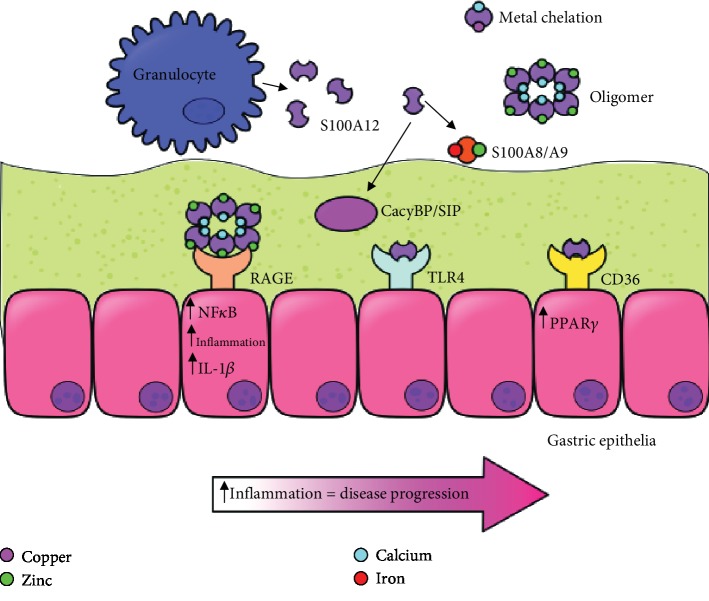
Conceptual diagram of the association of S100A12 in various digestive diseases and health. S100A12 (EN-RAGE or calgranulin C, depicted in a dimer form) is produced by innate immune cells such as granulocytes and participates in the chemotaxis of innate immune cells. It can exist as a dimer or oligomer and can bind divalent cations including zinc, copper, and calcium to promote “nutritional immunity” against invading microbial pathogens. S100A12 interacts with cell surface membranes as well as RAGE, TLR4, and CD36 receptors to promote proinflammatory signaling and disease progression. S100A12 also interacts with CacyBP/SIP and S100A9. S100A12 binding of calcium and zinc enhances oligomerization and interactions with receptors such as RAGE.

S100A12 has proinflammatory activity and is considered to be a key player in inflammation [27–33]. S100A12 is a damage-associated molecular pattern that alters immune function and leads to changes in a variety of cellular processes [27–33]. It acts as a chemotactic molecule and recruits both mast cells and monocytes to sites of inflammation [27–29]. S100A12 signals through the RAGE V receptor domain to induce cellular proliferation and proinflammatory signaling [30–34]. S100A12 interacts with membranes via specific lipid and ion-dependent interactions, including interactions with lipids in solution as well as interactions with lipid rafts from granulocytes [15, 31]. S100A12 binds more tightly to negatively charged lipids, and in the presence of calcium and zinc ions, conformational changes occur in S100A12 suggesting stabilization of charged residues within the protein structure [15, 25]. This, in turn, facilitates S100A12 interaction with membrane receptors, indicating levels of these ions within subcellular compartments could influence the translocation of S100A12 across membranes, a pathway that has been implicated for other members of this family such as S100A6 and S100A13 [23, 35, 36]. Once secreted, S100A12 exhibits cytokine-like activities which include proinflammatory signaling through several pathways (Figure 2) and antimicrobial activity [21, 23, 33, 34]. The majority of the antimicrobial activity of this protein is largely attributed to its ability to bind and chelate nutrient metals in a process known as “nutritional immunity.” Supplementation with exogenous sources of nutrient metals such as zinc can ablate the antimicrobial and antivirulence properties attributed to S100A12 [16, 37–39]. Additionally, S100A12 signals through the TLR4 pathway (Figure 2) and activates monocytes [30, 40]. The activation of TLR4 signaling by S100A12 leads to enhanced activation and migration of human monocytes and cognate upregulation of proinflammatory cytokines such as IL-1*β*, IL-6, and IL-8 [41]. Interestingly, administration of a TLR4 blocker significantly abrogated monocyte migration due to S100A12 activation, underscoring its importance in innate immune cell activation [41].

S100A12 is also a ligand for the receptor for advanced glycation end products (RAGE) on monocytes or epithelial cells [29, 32, 42–44]. S100A12 promotes NF-*κ*B activation and downstream upregulation of proinflammatory cytokines such as interleukin-1*β*, a cytokine that is implicated in numerous gastrointestinal diseases [45, 46]. There is emerging evidence that S100A12 interacts with CacyBP/SIP and S100A9 and also signals through the CD36 receptor (Figure 2), a class B scavenger receptor on some epithelial cells that acts as a fatty acid transporter [47, 48]. Interestingly, CD36 expression is tightly regulated by both the RAGE and TLR4 pathways; however, binding to CD36, RAGE, and TLR4 may be cell type- and tissue-specific and not necessarily overlapping signaling pathways induced by S100A12. Together, S100A12 interaction with these pathways conspires to increase proinflammatory immune signaling and promote inflammation as a consequence of [49]. A better understanding of these signaling axes and their role in pathogenesis could lead to novel molecular targets for chemotherapeutic interventions.

## 2. Methods

A scoping review of the literature was performed based on methods described by Arksey and O'Malley [50]. The central research question was “What is known from the existing literature about the association between S100A12 and digestive diseases?” The inclusion criteria for papers included English language and primary studies that evaluated S100A12 expression or activity associated with digestive disease including gastroenteritis, colitis, irritable bowel syndrome (IBS), inflammatory bowel disease (IBD), Crohn's disease, gastritis, or cancer.

Our search was conducted using PubMed with the search phrase “S100A12/calgranulin C/EN-RAGE AND structure, function, digestive disease, gastroenteritis, colitis, irritable bowel syndrome (IBS), inflammatory bowel disease (IBD), Crohn's disease, gastritis, cancer” with the last search performed on May 24, 2019. We identified additional studies in the references of articles identified in our primary search. A further review of the abstracts of these articles was performed to validate relevance and inclusion. Full articles were reviewed from this pool, and articles were excluded if the article was focused on other calgranulin proteins (such as calprotectin) or if full inclusion criteria could not be satisfied. The resulting manuscripts which support a link between S100A12 and specific gastrointestinal diseases are collated in Table 1. Additional manuscripts were incorporated in the process of peer review.

## 3. Results

### 3.1. S100A12 in Gastric Diseases

The expression of S100A12 has been examined in the setting of gastric cancer (GC). Comparing noncancerous gastric mucosa and tumor tissue, S100A12 was expressed in gastric epithelial cell lines and stromal cells (i.e., monocytes, lymphocytes, and neutrophils) in both conditions [51, 52]. The staining pattern showed stronger signal in stromal cells on cases and controls, with the nucleus and cytoplasm being clearly visible in stromal lines while only the cytoplasm stained positive in epithelial cells [51]. However, S100A12 expression was reduced in gastric cancer epithelia when compared to noncancerous gastric epithelial cells [52]. S100A12 mRNA analysis showed a decrease in expression in GC tissues when compared to noncancerous tissues. The authors detected a negative correlation between S100A12 expression in tumor cells and GC markers of severity, such as size, depth of invasion, TNM stage, Lauren classification, and tumor cell differentiation. Kaplan-Meier survival curves demonstrated an association between reduced expression of S100A12 in GC and worse survival outcomes. With these results, the authors suggest that calgranulin C may serve as a novel prognostic marker for detecting aggressive GC. Further research is needed to explore whether S100A12 has protective roles in tumorigenesis and how those mechanisms may operate [51, 52].

*H. pylori* infection is the single biggest risk factor associated with gastric cancer, and gastritis is a critical process in the precancerous signaling cascade [53, 54]. In order to understand the association of S100A12 with *H. pylori*-associated gastritis, one study investigated gastric mucosa tissue samples from 18 children and divided them in 3 groups: 6 children positive for *H. pylori* and gastritis (group 1), 6 children negative for both gastritis and *H. pylori* (group 2), and 6 children negative for *H. pylori* but positive for gastritis (group 3) [52]. Infection was determined through culture, histological assessment, or both. Serial formalin-fixed, paraffin-embedded sections of antral biopsies were stained for S100 proteins A8, A9, and S100A12; results showed that children have normal gastric mucosa (group 2) or were negative for *H. pylori*, but had gastritis (group 3) and had very few S100-positive cells. However, in group 1, researchers found prominent S100A12 cellular staining of the lamina propria, while the gastric epithelium was negative. Quantification of these differences found significantly more S100A12-positive cells in the gastric mucosa of *H. pylori+*/gastritis+children when compared to group 2 and group 3 [52]. This study also reported a direct correlation between S100A12-positive cells and gastritis scores, linking S100A12 expression to inflammation of gastric mucosa infected with *H. pylori* [52].

In a related study, researchers investigated the role of S100A12 in controlling growth and virulence of *Helicobacter pylori* [37, 38]. Congruent with the research above, gastric mucosa infected with *H. pylori* exhibited abundant S100A12 when compared to noninfected tissue, localizing primarily to polymorphonuclear cells in response to infection [37]. This research also showed that in the presence of 750 *μ*g/ml S100A12, *H. pylori* growth was repressed by 40% and viability was decreased when compared to cultures without S100A12 [37]. This antibacterial effect was dose-dependent as at 1000 *μ*g/ml S100A12, *H. pylori* growth was reduced by 51% and viability decreased compared to controls. Adding exogenous zinc to S100A12-supplemented cultures ameliorated these effects, indicating that zinc sequestration is key to S100A12's antibacterial activity [37, 38].

In addition to inhibiting *H. pylori* growth, S100A12 also appeared to inhibit *H. pylori*-induced host IL-8 secretion, which is dependent on the *cag* Type-4 Secretion System (*cag* T4SS). When *H. pylori* strain 7.13 was exposed to 500 *μ*g/ml of purified S100A12, IL-8 expression was reduced by 38% compared to the control (*p* = 0.047). Concomitantly, when 50 *μ*M exogenous zinc was added, *H. pylori* elicited robust IL-8 secretion from host cells. Moreover, the IL-8 signaling pathway was not affected by S100A12, since adding TNF-*α* in the presence of S100A12 resulted in normal IL-8 induction. Furthermore, S100A12 repressed *cag* T4SS-dependent changes in cell morphology, namely, the “hummingbird phenotype” of gastric cells. This scattered and elongated cell form is elicited when *H. pylori* cytotoxin CagA is translocated to host epithelial cells through the *cag* T4SS, which induces cytoskeletal rearrangements that may facilitate gastric tumor metastasis. In uninfected AGS cells, less than 2% demonstrated the hummingbird phenotype compared to 36% of *H. pylori*-infected AGS cells. When the bacterium was exposed to S100A12 prior to coculture with AGS cells, the hummingbird phenotype was induced in only 11% (*p* = 0.009) of cells, and this phenotype was completely reversed by the addition of exogenous zinc (50% hummingbird phenotype; *p* = 0.047). Finally, the authors found that S100A12 inhibits the biogenesis of the *cag* T4SS at the host-pathogen interface. Approximately 80% of bacteria cultured in medium without S100A12 had Cag pili present, with an average of 5 pili per cell. Meanwhile, *H. pylori* exposed to S100A12 formed <1 pilus per cell (*p* < 0.001) and fewer cells were piliated (17%, *p* < 0.001). As expected, the addition of exogenous zinc to culture reversed this phenotype [37, 38].

### 3.2. S100A12 in Colitis

A recent study found that humans infected by *Campylobacter jejuni* have a 2-fold increase in S100A12 in the feces when compared to uninfected controls (*p* = 0.0291) [39]. Exploring this in a ferret model, one of the few models used to validate *Campylobacter jejuni* infections and interactions within a vertebrate host, S100A12, was found to be increased in the feces of inoculated ferrets [39] with fecal samples exhibiting a similar 2-fold increase over control animals at 7 days postinfection (754.8 ± 110.8 pg/ml versus 376.6 ± 175.6 pg/ml, *p* < 0.05). Interestingly, as the concentrations of S100A12 peaked, the amount of viable *C. jejuni* in feces declined substantially. The authors also found that compared to uninfected ferrets, *C. jejuni*-infected ferrets had elevated levels of IL-10 and TNF-*α*, by 3-fold and 2-fold (*p* < 0.05), respectively, and populations of granulocytes and macrophages trafficked to and peaked in colonic tissue early in the infection course (day 3), followed by gradual resolution at day seven. To explore the negative correlation between S100A12 levels and viable *C. jejuni* in feces, the authors treated *C. jejuni* cultures with S100A12, finding that bacterial growth was significantly reduced compared to untreated controls. Moreover, when S100A12-treated *C. jejuni* cultures were supplemented with zinc, growth increased compared to unsupplemented cultures (*p* < 0.01). This showed that S100A12-dependent inhibition of *C. jejuni* occurs through sequestration of zinc, similar to findings described in the related Epsilonproteobacterium, *H. pylori*. Surprisingly, transcriptome analysis of S100A12-treated *C. jejuni* cultures did not find increased transcription of zinc transport systems when compared to control cultures. RNAseq analysis revealed 5 clusters of orthologous groups (COGS) that exhibited significant transcript increases; energy production and conversion genes represented the overwhelming majority (*p* < 0.05). However, 4 COGS showed reduced gene transcription in the treatment group, and more than 50% of these genes were within groups responsible for translation and ribosomal structure/biogenesis (*p* < 0.05) [39].

Another interesting study evaluated fecal S100A12 in the setting of necrotizing enterocolitis (NEC) in Extremely Low Birthweight (ELBW, <1000 g) infants and found that in the 5 days prior to NEC symptom onset, there was a steep 9.8-fold rise in fecal median total bacterial CFU/g counts and a 21.6-fold rise in fecal median *E. coli* CFU/g counts, with *p* < 0.05 and *p* < 0.001, respectively [55]. Moreover, the investigators found that fecal samples had a positive, albeit weak, Pearson's correlation between S100A12 and total bacterial CFU/g feces (*r*^2^ = 0.40, *p* < 0.01) and *E. coli* CFU/g feces (*r*^2^ = 0.40, *p* < 0.01) [55]. This suggests that S100A12 might be used as a noninvasive biomarker to help predict NEC, a significant cause of morbidity and mortality in ELBW infants [55–58]. However, a significant limitation of this study was the small number of patients enrolled (*n* = 68). A subsequent study revealed that fecal S100A12 concentrations were elevated concomitant with NEC disease progression and that multiple pathogenic bacteria were associated with this disease progression [55, 56]. Additional studies by Däbritz and colleagues attempted to determine if longitudinal measurements of fecal S100A12 could detect Very Low Birth Weight (VLBW) infants at risk for intestinal distress apart from NEC. Their results indicated that median levels of fecal S100A12 were significantly higher in patients with intestinal distress both before and at onset of disease compared with unaffected reference infants. Median levels of fecal S100A12 declined steadily to baseline levels within 2 weeks of disease onset. Their study concluded that the ideal cutoff value for identifying patients with intestinal distress within 7 days before disease onset was 60 *μ*g/kg (sensitivity 0.73; specificity 0.55) [59]. A companion study by the same group determined that gestational age and birth weight were significantly lower in the patients with NEC compared with unaffected reference infants and that fecal S100A12 levels were significantly higher in patients with severe NEC at onset of disease. This study also determined that S100A12 levels were significantly higher at 4-10 days before onset of NEC compared with unaffected reference infants (ideal cutoff value, 65 *μ*g/kg; sensitivity, 0.76; specificity, 0.56), a result that was not seen with the related protein, calprotectin [60]. Together, these results indicate that S100A12 could be an important marker of intestinal inflammation utilized to identify at risk populations for NEC. Further research is required to confirm and expand these findings.

### 3.3. S100A12 in IBD (including Crohn's and Ulcerative Colitis) and IBS

Inflammatory bowel disease (IBD) and irritable bowel syndrome (IBS) are two distinct gastrointestinal disorders. The former (IBD) is characterized by chronic inflammation, ulcers, and lesions within the gastrointestinal tract and includes disorders such as Crohn's and ulcerative colitis. The latter (IBS) is noninflammatory and is not associated with lesions or ulcers of the bowel and often involves the colon [61]. S100A12 has been studied in the setting of IBD and IBS and is gaining appreciation as a biomarker that could be used to distinguish between these disorders [61, 62]. In one article, authors used a previously described prospective, randomized controlled trial cohort from the POCER study to assess postoperative recurrence under endoscopic and fecal biomarker scrutiny. Ileocolonoscopy was done in 2/3 of patients at 6 months and all patients at 18 months after ileocolonic resection. Remission was defined as Rutgeerts score i0 or i1, and disease flare-ups were defined as scores between i2 and i4. Stool samples were collected preoperatively and at 6, 12, and 18 months postoperatively from 174 patients at 17 hospitals in New Zealand and Australia, and levels of fecal calprotectin (FC), fecal lactoferrin (FL), and fecal S100A12 (FS) were determined. S100A12 measurements for monitoring response to treatment step-up did not reach statistical significance, while the other biomarkers performed significantly better [62].

Another study using capsule endoscopy (CE) and fecal biomarkers in small-bowel CD compared FC, FL, and FS to assess remission and predict relapse. The study included 43 patients from Australian academic hospitals between 18 and 70 years of age with small bowel involvement deemed in remission by a CDAI score (<150). Patients underwent baseline CE and were followed prospectively for 12 months, or until a clinical flare. Baseline and endpoint fecal biomarkers were assessed. Although a positive correlation existed between Capsule Endoscopy Scoring Index (CESI, Lewis score) and baseline fecal biomarkers, fecal calprotectin and fecal lactoferrin consistently performed better than fecal S100A12. Regarding clinical flare detection, 14% [6] of patients had a relapse during the 12-month follow-up period (CDAI > 220), at a median of 7 months. Of these patients, 83% [5] had increased fecal calprotectin and lactoferrin at baseline while only 50% exhibited elevated S100A12 levels. Endpoint markers at flare-up were available for 5 out of 6 relapsed patients and showed that fecal calprotectin was increased in all 5, FL in 4 out of 5, and FS in only 2 out of 5 patients. Therefore, calprotectin and lactoferrin seem to perform better as a biomarker to track small bowel CD than S100A12 [63].

Studies converge in the potential use of calgranulin C as a biomarker to differentiate between inflammatory bowel disease (IBD) and inflammatory bowel syndrome (IBS) [64–66]. Researchers in Germany found that fecal S100A12 rises in IBD when compared to IBS or healthy controls, with no significant difference between CD and UC detected. Fecal levels in IBD were 2.45 ± 1.15 mg/kg compared with healthy controls at 0.006 ± 0.03 mg/kg (*p* < 0.001) or IBS patients 0.05 ± 0.11 mg/kg (*p* < 0.001). S100A12 was found to be more granulocyte-specific and did not rise in cases of viral gastroenteritis but did rise with bacterial gastroenteritis. Calprotectin, a more established biomarker for tracking gut inflammation, increased in the setting of both viral and bacterial gastroenteritis; thus, the authors found fecal S100A12 to be more specific than S100A8/A9. Although the article published excellent sensitivity and specificity for S100A12 to distinguish active IBD from inactive IBD, the study had poor case-control matching and heterogeneous groups, making comparisons difficult. Nonetheless, the authors reported that S100A12 levels and histology inflammation score correlated significantly in both UC and CD, but only UC showed a significant correlation with the clinical CAI score (*r* = 0.415, *p* < 0.05) [66].

Another article demonstrated S100A12's ability to significantly distinguish between active and inactive IBD vs. IBS. In contrast, this article did not detect a significant difference between S100A12 serum levels in active versus inactive IBD (*p* = 0.546) [61]. A further study evaluating fecal S100A12 and management of IBD also detected a significant difference between IBD and IBS [61]. The S100A12 median (IQR) for patients with IBD was 69.8 *μ*g/g versus 0.7 *μ*g/g for IBS (*p* < 0.001). Levels for UC and CD were not statistically different (*p* = 0.246), demonstrating that fecal S100A12 did not differentiate between forms of IBD, but using a ROC curve cut-off value of 2.8 *μ*g/g, fecal S100A12 discriminated between IBD and IBS, similar to fecal calprotectin and previous S100A12 findings [66, 67]. This study also found that fecal levels of S100A12 moderately correlated with the Mayo UC severity score (*r* = 0.687; *p* = 0.001) but did not correlate with the CD Harvey-Bradshaw disease index (*r* = 0.259; *p* = 0.392). This suggests that fecal S100A12 levels mirror disease severity scores in UC, but may not apply to CD.

Carefully designed, large, prospective studies on the discriminative ability of S100A12 are scarce, and an absence of test standardization plagues research in the field leading to inconsistency and lack of reproducibility between research groups. One study, however, attempted to overcome such limitations by using the previously described CACATU (Calprotectin or Calgranulin C Test before Undergoing endoscopy) cohort from the Netherlands and Belgium [65]. This multicenter, delayed-type, cross-sectional diagnostic accuracy study recruited 354 children between 6 and 17 years of age, from 16 secondary and 3 tertiary level hospitals. Baseline characteristics were taken, and fecal samples were collected for biomarker and pathogen assessment; a study algorithm was followed to standardize the assignment of patients to either endoscopy or clinical follow-up, with Bayesian corrections performed to avoid differential verification bias. When using common thresholds for calprotectin, S100A12 performed significantly better in terms of specificity. When ROC-based optimal cut-offs were set with S100A12 at 0.75 *μ*g/g and calprotectin at 400 *μ*g/g, both tests perform equally well at predicting IBD and guiding endoscopy. Clinically, however, the calprotectin test required a two-threshold strategy while S100A12 result interpretation was more binary, making it more useful in triaging children with potential IBD to endoscopy [65].

S100 proteins have also been examined for usefulness as transcriptional blood biomarkers to determine mucosal healing. One study of 152 patients collected whole blood at the time of endoscopy [68]. Gene transcripts that correlated with inflammation at endoscopy were identified using RT-PCR and then validated in an independent group of 111 UC patients with active disease (*n* = 86) or in remission (*n* = 25). The first group had 25 UC patients with active disease (*n* = 17) and in remission (*n* = 8), versus 20 non-IBD controls; this cohort had their blood analyzed using microarrays. The second group, totaling 16 UC patients that received anti-TNF*α* treatment (infliximab, adalimumab, and golimumab), was followed for 14 weeks, with pretreatment/posttreatment gene expression and mucosal healing scores analyzed for correlation [62]. The authors found that 122 genes were altered in the blood of active UC patients when compared to remission and control patients, with 80% of them being upregulated. Overall, the changes were unimpressive, with only 15 genes showing a greater than 2-fold change compared to remission and control patients [68]. These genes were likely neutrophil-derived, with genes including CD177, haptoglobin (HP), G-protein coupled receptor 84 (GPR84), hexokinase 3 (HK3), arginase 1 (ARG1), annexin A3 (ANXA3), and calgranulin C (S100A12). The authors found that S100A12 transcripts in the blood had a significant but weak Spearman's rank correlation (rho = 0.31; *p* < 0.01) and revealed that the test could only detect differences between Mayo score 0 (no inflammation) and Mayo score 3 (severe inflammation). Other markers, like CD177 and haptoglobin, performed significantly better. However, the authors found that S100A12 decreased after 14 weeks of anti-TNF*α* treatment and correlated significantly with the Modified Score (MS; *p* = 0.009), performing similar to other biomarkers analyzed [68].

In summary, these studies suggest that fecal S100A12 could be utilized in IBD as an indicator of mucosal healing [69, 70] and a predictor of relapse [71–76]. A multicenter comparison of predictive outcomes and response monitoring suggest S100A12 could also be a predictive marker for severe ulcerative colitis in [72]. However, additional longitudinal studies with adequate statistical power to evaluate both adults and children are needed in order to confirm or refute these findings and dispel apparent inconsistencies in the outcomes. However, from the studies cited above, it seems that fecal S100A12 outperforms its serum measurements, probably due to granulocyte infiltration into inflamed intestinal mucosa and subsequent cellular debris sloughing off into the lumen. Furthermore, the best use of S100A12 seems to be in the scenario where providers need to triage, noninvasively, adults and children with IBD whom would require endoscopy versus IBS patients whom do not need to be scoped for a benign, functional disorder. In this scenario, S100A12 performs slightly better than calprotectin, when one considers the most frequently used cut-off values. This increased specificity might be due to S100A12 being secreted mostly by activated granulocytes, whereas calprotectin is secreted by neutrophils, monocytes, and epithelial cells. S100A12 does not discriminate between UC and CD and weakly correlates to clinical and histological severity scores in UC, but not in CD. This shows that S100A12 may not be the best marker to predict relapse and monitor response to treatment in IBD, with calprotectin still being superior in this setting. Finally, research using transcriptional biomarkers shows promise, although blood S100A12 underperformed in comparison to others, like haptoglobin or CD177. Future research will elucidate the feasibility of fecal S100A12 transcriptional biomarkers and its usefulness compared to more established, noninvasive markers [68]. In summary, the above data indicate that the best clinical use of S100A12 is in the differentiation between IBD and IBS, with fecal S100A12 outperforming serum S100A12 measurements.

### 3.4. S100A12 in Colon Cancer

The strategy used for this review found few articles that specifically pertain to S100A12 and colon cancer (CC). A proteomic analysis comparing malignant human colonic tissue to healthy control tissues or clinical controls (GI disorders other than CC) using 2-D-LC-ESI-MS identified 484 upregulated proteins in CC, including S100A12 [77]. Of these upregulated proteins, 84.7% were found in both tumor and control samples, while 10.7% and 4.6% were found exclusively in control and cancer tissues, respectively. Furthermore, S100A12 serum levels were markedly elevated in CC patients compared to healthy (median 139 ng/ml versus 39 ng/ml; *p* < 0.0001) and clinical controls (139 ng/ml versus 80 ng/ml; *p* < 0.0001). Most importantly, the authors compared the performance of S100A12 with another established marker used to screen for CC, namely, serum CEA. The relationship between sensitivity and specificity of CEA and S100A12 to detect malignancy was represented by ROC curves. The AUROC result was 0.87 for S100A12 compared to 0.74 for CEA when malignant samples were compared with healthy controls, and 0.66 versus 0.74 when comparing CC patients to clinical controls. Therefore, although S100A12 performs better than CEA at discriminating between colon cancer and healthy patients, it is not able to detect differences when other gastrointestinal disorders confound the results. In this instance, CEA maintains higher overall performance than S100A12, which may be due to the elevation of S100A12 in the serum during other inflammatory conditions, described above [77]. Further research is needed to determine if fecal proteomics or transcriptomics perform better than serum measurements when colonic cancer is investigated, and the association of S100A12 with colon cancer could be leveraged as a putative biomarker for disease detection.

### 3.5. S100A12 as a Chemotherapeutic Target

The receptor for advanced glycation end products (RAGE) and its ligands have been described as a novel pathway connecting the innate immune system with inflammatory responses. S100A12 is one of the ligands that activates RAGE, producing downstream signaling that involves key mediators such as NF-*κ*B, MAP kinase, CD36, TLRs, and other molecules [29, 30, 32, 34, 43, 45, 47, 48]. Because S100A12 is involved in many different diseases, such as Juvenile Rheumatoid Arthritis (JRA), asthma, Behçet's, Kawasaki disease, and IBD, it is natural that researchers would attempt to target this interaction pharmacologically [42, 78–81]. As described above, S100A12 participates in what has been called “nutritional immunity” and theoretically could be used to diminish the concentration of essential factors for bacterial growth in the GI lumen (like zinc) [37–39]. This approach would have to take into consideration the restoration effect noted in many of the above studies, where zinc supplementation causes a rebound in bacterial growth despite initial S100A12-dependent inhibition of bacterial growth. This effect is relevant in clinical settings, since many patients use over the counter vitamins and supplements, often containing zinc in their formulations. Another potential approach would be to block RAGE through competitive inhibitors [30]. Our search did not find any specific applications of this strategy in digestive diseases, but a study using a chemically induced asthma model in mice found that a RAGE antagonist peptide (RAP) successfully blunted airway reactivity, inflammation, goblet cell metaplasia, and decreased Th2 cytokines [79]. As a result, this study suggests that there may be a value in examining this approach for a variety of gastrointestinal diseases.

Lastly, our review encountered robust literature on the association of S100A12 with atherosclerosis, a well-known systemic inflammatory disease that may affect abdominal arteries. In this context, patients present with either acute or chronic mesenteric ischemia. Here, the connection between intestinal ischemia, coronary artery disease (CAD), plaque instability, and atherosclerosis cannot be overstated. In the Rotterdam study, a prospective population-based investigation of 839 participants without CAD being followed for 10.6 years identified S100A12 elevation in the highest tertile as having 2.6-fold higher risk of developing CAD compared with participants in the lowest tertile (hazard ratio, 2.59; 95% CI 1.52-4.40) [82]. Another study found that in autopsied sudden cardiac death victims, S100A12 and RAGE expression was enhanced in macrophages and smooth muscle cells in ruptured coronary artery plaques, indicating a potential role in plaque vulnerability [83]. Moreover, Q-compound ABR-215757 (Paquinimod) was found to bind *in vitro* with S100A12 and reduce atherosclerotic lesion complexity in transgenic mice, demonstrating that direct inhibition of S100A12 can be achieved without affecting RAGE, a multiligand receptor [84]. Finally, “leaky gut” and its chronic inflammation backdrop are increasingly recognized as major factors in many gastrointestinal diseases. Additionally, S100A12 has been exploited to block S100A9 binding to RAGE V domain, indicating S100A12 itself could potentially be utilized as a chemotherapeutic substrate [85]. Hypothetically, one can imagine that blocking the S100A-family proteins/RAGE interactions may reduce inflammation in the gut and in other organs and systems.

## 4. General Discussion

S100A12 is a multifunctional host protein that participates in several biological pathways. It is constitutively expressed by innate immune cells such as neutrophils and is inducibly expressed by a variety of cell types including epithelial cells [16]. S100A12 is secreted by granulocytes via ROS and potassium homeostasis pathways during acute innate immune cell responses [8] and subsequently induces a variety of antimicrobial and immunoregulatory phenotypes (see Figure 2). S100A12 can bind a variety of inorganic ions including calcium and transition metals such as zinc and copper [19–22]. This ion-binding activity influences both the quaternary structure of S100A12 and also its activity [25, 26]. In the context of infection, S100A12 metal binding is responsible for starving invading pathogens of critical micronutrients involved in cellular processes such as respiration, cell division, and virulence factor deployment [37–39], thereby inhibiting microbial growth, proliferation, and disease progression as an innate immune strategy. In the context of immunoregulation, S100A12 has several targets which promote interactions that alter the host immune response. S100A12 binds to and activates the RAGE receptor, which can lead to increased NF-*κ*B activation, proinflammatory signaling, and the initiation of inflammation responses [9, 10, 28, 30, 32, 40, 43, 85]. S100A12 also binds to the TLR-4 receptor to promote proinflammatory cytokine secretion and inflammatory responses [43, 44]. Additionally, S100A12 directly interacts with both S100A9 [10] and CacyBP/SIP [48]. Because the former also signals through the RAGE receptor pathway, this interaction could potentially alter downstream RAGE signaling. CacyBP/SIP is involved in ubiquitinylation and beta-catenin degradation; thus, it is possible that S100A12 could influence these activities through binding.

## 5. Conclusions

In conclusion, we report that elevated levels of S100A12 are associated with gastroenteritis, necrotizing enterocolitis, gastritis, gastric cancer, Crohn's disease, irritable bowel syndrome, inflammatory bowel disease, and digestive tract cancers. Together, these results reveal S100A12 is an important molecule broadly associated with the pathogenesis of digestive diseases and that S100A12 could be a potential biomarker for early diagnosis or a target for chemotherapeutic intervention.

## Figures and Tables

**Table 1:** Digestive diseases and their association with S100A12.

Digestive disease	Association with S100A12	References
Atrophic gastritis	Elevated in gastritis samples compared to healthy controls	[52, 53]
Gastric cancer	Decreased in GC samples compared to noncancerous samples	[51]
Necrotizing enterocolitis	Elevated in NEC samples with respect to disease progression	[45–50]
Colitis	Elevated in colitis samples compared to healthy controls	[55, 56]
Colon cancer	Elevated serum S100A12 in CC patients vs. healthy controls	[73]
Irritable bowel syndrome	Elevated in flares	[61, 62]
Inflammatory bowel disease	Elevated in active disease	[37, 59–71]
Crohn's disease	Elevated in Crohn's samples vs. healthy controls	[57, 58, 63–65]
Gastrointestinal surgical injury	Elevated in injured patients	[33]

## References

[1] Donato R., Cannon B. R., Sorci G. (2013). Functions of S100 proteins. *Current Molecular Medicine*.

[2] Heizmann C. W., Fritz G., Schäfer B. W. (2002). S100 proteins: structure, functions and pathology. *Frontiers in Bioscience*.

[3] van Lent P. L., Grevers L. C., Schelbergen R. (2010). S100A8 causes a shift toward expression of activatory Fc*γ* receptors on macrophages via toll-like receptor 4 and regulates Fc*γ* receptor expression in synovium during chronic experimental arthritis. *Arthritis and Rheumatism*.

[4] Donato R. (2003). Intracellular and extracellular roles of S100 proteins. *Microscopy Research and Technique*.

[5] Ravasi T., Hsu K., Goyette J. (2004). Probing the S100 protein family through genomic and functional analysis. *Genomics*.

[6] Vogl T., Pröpper C., Hartmann M. (1999). S100A12 is expressed exclusively by granulocytes and acts independently from MRP8 and MRP14. *The Journal of Biological Chemistry*.

[7] Bagheri V. (2017). S100A12: friend or foe in pulmonary tuberculosis?. *Cytokine*.

[8] Tardif M. R., Chapeton-Montes J. A., Posvandzic A., Pagé N., Gilbert C., Tessier P. A. (2015). Secretion of S100A8, S100A9, and S100A12 by neutrophils involves reactive oxygen species and potassium efflux. *Journal of Immunology Research*.

[9] Khan M. I., Su Y. K., Zou J., Yang L. W., Chou R. H., Yu C. (2018). S100B as an antagonist to block the interaction between S100A1 and the RAGE V domain. *PLoS One*.

[10] Chang C. C., Khan I., Tsai K. L. (2016). Blocking the interaction between S100A9 and RAGE V domain using CHAPS molecule: a novel route to drug development against cell proliferation. *Biochimica et Biophysica Acta (BBA) - Proteins and Proteomics*.

[11] Gupta A. A., Chou R. H., Li H., Yang L. W., Yu C. (2013). Structural insights into the interaction of human S100B and basic fibroblast growth factor (FGF2): effects on FGFR1 receptor signaling. *BBA Proteins and Proteomics*.

[12] Dell'Angelica E. C., Schleicher C. H., Santomé J. A. (1994). Primary structure and binding properties of calgranulin C, a novel S100-like calcium-binding protein from pig granulocytes. *The Journal of Biological Chemistry*.

[13] Wicki R., Marenholz I., Mischke D., Schäfer B. W., Heizmann C. W. (1996). Characterization of the human S100A12 (calgranulin C, p6, CAAF1, CGRP) gene, a new member of the S100 gene cluster on chromosome 1q21. *Cell Calcium*.

[14] Hatakeyama T., Okada M., Shimamoto S., Kubota Y., Kobayashi R. (2004). Identification of intracellular target proteins of the calcium-signaling protein S100A12. *European Journal of Biochemistry*.

[15] Garcia A. F., Lopes J. L., Costa-Filho A. J., Wallace B. A., Araujo A. P. (2013). Membrane interactions of S100A12 (calgranulin C). *PLoS One*.

[16] Goyette J., Geczy C. L. (2011). Inflammation-associated S100 proteins: new mechanisms that regulate function. *Amino Acids*.

[17] Moroz O. V., Antson A. A., Murshudov G. N. (2001). The three-dimensional structure of human S100A12. *Acta Crystallographica. Section D: Biological Crystallography*.

[18] Moroz O. V., Dodson G. G., Wilson K. S., Lukanidin E., Bronstein I. B. (2003). Multiple structural states of S100A12: A key to its functional diversity. *Microscopy Research and Technique*.

[19] Moroz O. V., Blagova E. V., Wilkinson A. J., Wilson K. S., Bronstein I. B. (2009). The crystal structures of human S100A12 in apo form and in complex with zinc: new insights into S100A12 oligomerisation. *Journal of Molecular Biology*.

[20] Moroz O. V., Antson A. A., Grist S. J. (2003). Structure of the human S100A12-copper complex: implications for host-parasite defence. *Acta Crystallographica. Section D, Biological Crystallography*.

[21] Hung K. W., Hsu C. C., Yu C. (2013). Solution structure of human Ca (2+)-bound S100A12. *Journal of Biomolecular NMR*.

[22] Moroz O. V., Burkitt W., Wittkowski H. (2009). Both Ca^2+^ and Zn^2+^ are essential for S100A12 protein oligomerization and function. *BMC Biochemistry*.

[23] Garcia A. F., Garcia W., Nonato M. C., Araújo A. P. (2008). Structural stability and reversible unfolding of recombinant porcine S100A12. *Biophysical Chemistry*.

[24] Wang Q., Aleshintsev A., Bolton D., Zhuang J., Brenowitz M., Gupta R. (2019). Ca (II) and Zn (II) cooperate to modulate the structure and self-assembly of S100A12. *Biochemistry*.

[25] Reis R. A., Bortot L. O., Caliri A. (2014). *In silico* assessment of S100A12 monomer and dimer structural dynamics: implications for the understanding of its metal-induced conformational changes. *Journal of Biological Inorganic Chemistry*.

[26] Streicher W. W., Lopez M. M., Makhatadze G. I. (2010). Modulation of quaternary structure of S100 proteins by calcium ions. *Biophysical Chemistry*.

[27] Yan W. X., Armishaw C., Goyette J. (2008). Mast cell and monocyte recruitment by S100A12 and its hinge domain. *The Journal of Biological Chemistry*.

[28] Miranda L. P., Tao T., Jones A. (2001). Total chemical synthesis and chemotactic activity of human S100A12 (EN-RAGE). *FEBS Letters*.

[29] Hofmann Bowman M. A., Heydemann A., Gawdzik J., Shilling R. A., Camoretti-Mercado B. (2011). Transgenic expression of human S100A12 induces structural airway abnormalities and limited lung inflammation in a mouse model of allergic inflammation. *Clinical & Experimental Allergy*.

[30] Chiou J. W., Fu B., Chou R. H., Yu C. (2016). Blocking the Interactions between calcium-bound S100A12 protein and the V domain of RAGE using tranilast. *PLoS One*.

[31] Nacken W., Sorg C., Kerkhoff C. (2004). The myeloid expressed EF-hand proteins display a diverse pattern of lipid raft association. *FEBS Letters*.

[32] Xie J., Burz D. S., He W., Bronstein I. B., Lednev I., Shekhtman A. (2007). Hexameric calgranulin C (S100A12) binds to the receptor for advanced glycated end products (RAGE) using symmetric hydrophobic target-binding patches. *The Journal of Biological Chemistry*.

[33] Moroz O. V., Antson A. A., Dodson E. J. (2002). The structure of S100A12 in a hexameric form and its proposed role in receptor signalling. *Acta Crystallographica. Section D, Biological Crystallography*.

[34] Rouleau P., Vandal K., Ryckman C. (2003). The calcium-binding protein S100A12 induces neutrophil adhesion, migration, and release from bone marrow in mouse at concentrations similar to those found in human inflammatory arthritis. *Clinical Immunology*.

[35] Hsieh H. L., Schäfer B. W., Cox J. A., Heizmann C. W. (2002). S100A13 and S100A6 exhibit distinct translocation pathways in endothelial cells. *Journal of Cell Science*.

[36] Goebeler M., Roth J., Van den Bos C., Ader G., Sorg C. (1995). Increase of calcium levels in epithelial cells induces translocation of calcium-binding proteins migration inhibitory factor related protein 8 (MRP8) and MRP14 to keratin intermediate filaments. *The Biochemical Journal*.

[37] Haley K. P., Delgado A. G., Piazuelo M. B. (2015). The human antimicrobial protein calgranulin C participates in control of helicobacter pylori growth and regulation of virulence. *Infection and Immunity*.

[38] Jackson E., Little S., Franklin D. S., Gaddy J. A., Damo S. M. (2017). Expression, purification, and antimicrobial activity of S100A12. *Journal of Visualized Experiments*.

[39] Shank J. M., Kelley B. R., Jackson J. W. (2018). Participates in the control of *Campylobacter jejuni* growth via zinc sequestration. *Infection and Immunity*.

[40] Huang S. M., Chang Y. H., Chao Y. C. (2013). EGCG-rich green tea extract stimulates sRAGE secretion to inhibit S100A12-RAGE axis through ADAM10-mediated ectodomain shedding of extracellular RAGE in type 2 diabetes. *Molecular Nutrition & Food Research*.

[41] Foell D., Wittkowski H., Kessel C. (2013). Proinflammatory S100A12 can activate human monocytes via toll-like receptor 4. *American Journal of Respiratory and Critical Care Medicine*.

[42] Foell D., Wittkowski H., Ren Z. (2008). Phagocyte-specific S100 proteins are released from affected mucosa and promote immune responses during inflammatory bowel disease. *The Journal of Pathology*.

[43] Kovačić M., Mitrović-Ajtić O., Beleslin-Čokić B. (2018). TLR4 and RAGE conversely mediate proinflammatory S100A8/9-mediated inhibition of proliferation-linked signaling in myeloproliferative neoplasms. *Cellular Oncology*.

[44] Loes A. N., Bridgham J. T., Harms M. J. (2018). Coevolution of the toll-like receptor 4 complex with calgranulins and lipopolysaccharide. *Frontiers in Immunology*.

[45] Kang J. H., Hwang S. M., Chung I. Y. (2015). S100A8, S100A9 and S100A12 activate airway epithelial cells to produce MUC5AC via extracellular signal-regulated kinase and nuclear factor *κ*B pathways. *Immunology*.

[46] McEntee C. P., Finlay C. M., Lavelle E. C. (2019). Divergent roles for the IL-1 family in gastrointestinal homeostasis and inflammation. *Frontiers in Immunology*.

[47] Farokhzadian J., Mangolian Shahrbabaki P., Bagheri V. (2019). S100A12-CD36 axis: a novel player in the pathogenesis of atherosclerosis?. *Cytokine*.

[48] Filipek A., Jastrzebska B., Nowotny M., Kuznicki J. (2002). CacyBP/SIP, a calcyclin and Siah-1-interacting protein, binds EF-hand proteins of the S100 family. *The Journal of Biological Chemistry*.

[49] Pietzsch J., Hoppmann S. (2009). Human S100A12: a novel key player in inflammation?. *Amino Acids*.

[50] Arksey H., O'Malley L. (2005). Scoping studies: towards a methodological framework. *International Journal of Social Research Methodology*.

[51] Li D., Zeng Z., Yu T. (2016). Expression and clinical implication of S100A12 in gastric carcinoma. *Tumor Biology*.

[52] Leach S. T., Mitchell H. M., Geczy C. L., Sherman P. M., Day A. S. (2008). S100 calgranulin proteins S100A8, S100A9 and S100A12 are expressed in the inflamed gastric mucosa of *Helicobacter pylori*-infected children. *Canadian Journal of Gastroenterology*.

[53] Polk D. B., Peek R. M. (2010). *Helicobacter pylori:* gastric *cancer and beyond*. *Cancer*.

[54] Correa P., Piazuelo M. B. (2012). The gastric precancerous cascade. *Journal of Digestive Diseases*.

[55] Jenke A. C., Postberg J., Mariel B. (2013). S100A12 and hBD2 correlate with the composition of the fecal microflora in ELBW infants and expansion of *E. coli* is associated with NEC. *BioMed Research International*.

[56] Leach S. T., Lui K., Naing Z., Dowd S. E., Mitchell H. M., Day A. S. (2015). Multiple opportunistic pathogens, but not pre-existing inflammation, may be associated with necrotizing enterocolitis. *Digestive Diseases and Sciences*.

[57] Nantais-Smith L., Kadrofske M. (2015). Noninvasive biomarkers of necrotizing enterocolitis. *The Journal of Perinatal & Neonatal Nursing*.

[58] Robinson J. R., Rellinger E. J., Hatch L. D. (2017). Surgical necrotizing enterocolitis. *Seminars in Perinatology*.

[59] Däbritz J., Foell D., Wirth S., Jenke A. (2013). Fecal S100A12: identifying intestinal distress in very-low-birth-weight infants. *Journal of Pediatric Gastroenterology and Nutrition*.

[60] Däbritz J., Jenke A., Wirth S., Foell D. (2012). Fecal phagocyte-specific S100A12 for diagnosing necrotizing enterocolitis. *The Journal of Pediatrics*.

[61] Manolakis A. C., Kapsoritakis A. N., Georgoulias P. (2010). Moderate performance of serum S100A12, in distinguishing inflammatory bowel disease from irritable bowel syndrome. *BMC Gastroenterology*.

[62] De Cruz P., Kamm M. A., Hamilton A. L. (2015). Efficacy of thiopurines and adalimumab in preventing Crohn's disease recurrence in high-risk patients - a POCER study analysis. *Alimentary Pharmacology & Therapeutics*.

[63] Aggarwal V., Day A. S., Connor S. (2017). Role of capsule endoscopy and fecal biomarkers in small-bowel Crohn's disease to assess remission and predict relapse. *Gastrointestinal Endoscopy*.

[64] Di Ruscio M., Vernia F., Ciccone A., Frieri G., Latella G. (2017). Surrogate fecal biomarkers in inflammatory bowel disease: rivals or complementary tools of fecal calprotectin?. *Inflammatory Bowel Diseases*.

[65] Heida A., van de Vijver E., van Ravenzwaaij D. (2018). Predicting inflammatory bowel disease in children with abdominal pain and diarrhoea: calgranulin-C versus calprotectin stool tests. *Archives of Disease in Childhood*.

[66] Kaiser T., Langhorst J., Wittkowski H. (2007). Faecal S100A12 as a non-invasive marker distinguishing inflammatory bowel disease from irritable bowel syndrome. *Gut*.

[67] Whitehead S. J., Ford C., Gama R. M. (2017). Effect of faecal calprotectin assay variability on the management of inflammatory bowel disease and potential role of faecal S100A12. *Journal of Clinical Pathology*.

[68] Planell N., Masamunt M. C., Leal R. F. (2017). Usefulness of transcriptional blood biomarkers as a non-invasive surrogate marker of mucosal healing and endoscopic response in ulcerative colitis. *Journal of Crohn's & Colitis*.

[69] Boon G. J., Day A. S., Mulder C. J., Gearry R. B. (2015). Are faecal markers good indicators of mucosal healing in inflammatory bowel disease?. *World Journal of Gastroenterology*.

[70] Däbritz J., Langhorst J., Lügering A. (2013). Improving relapse prediction in inflammatory bowel disease by neutrophil-derived S100A12. *Inflammatory Bowel Diseases*.

[71] Turner D., Leach S. T., Mack D. (2010). Faecal calprotectin, lactoferrin, M2-pyruvate kinase and S100A12 in severe ulcerative colitis: a prospective multicentre comparison of predicting outcomes and monitoring response. *Gut*.

[72] Kopylov U., Rosenfeld G., Bressler B., Seidman E. (2014). Clinical utility of fecal biomarkers for the diagnosis and management of inflammatory bowel disease. *Inflammatory Bowel Diseases*.

[73] van de Logt F., Day A. S. (2013). S100A12: a noninvasive marker of inflammation in inflammatory bowel disease. *Journal of Digestive Diseases*.

[74] Judd T. A., Day A. S., Lemberg D. A., Turner D., Leach S. T. (2011). Update of fecal markers of inflammation in inflammatory bowel disease. *Journal of Gastroenterology and Hepatology*.

[75] Sherwood R. A. (2012). Faecal markers of gastrointestinal inflammation. *Journal of Clinical Pathology*.

[76] Manolakis A. C., Kapsoritakis A. N., Tiaka E. K., Potamianos S. P. (2011). Calprotectin, calgranulin C, and other members of the s100 protein family in inflammatory bowel disease. *Digestive Diseases and Sciences*.

[77] Thierolf M., Hagmann M. L., Pfeffer M. (2008). Towards a comprehensive proteome of normal and malignant human colon tissue by 2-D-LC-ESI-MS and 2-DE proteomics and identification of S100A12 as potential cancer biomarker. *Proteomics. Clinical Applications*.

[78] Foell D., Wittkowski H., Hammerschmidt I. (2004). Monitoring neutrophil activation in juvenile rheumatoid arthritis by S100A12 serum concentrations. *Arthritis and Rheumatism*.

[79] Yao L., Zhao H., Tang H. (2016). The receptor for advanced glycation end products is required for *β*-catenin stabilization in a chemical-induced asthma model. *British Journal of Pharmacology*.

[80] Han E. C., Cho S. B., Ahn K. J. (2011). Expression of pro-inflammatory protein S100A12 (EN-RAGE) in Behçet's disease and its association with disease activity: a pilot study. *Annals of Dermatology*.

[81] Ye F., Foell D., Hirono K. I. (2004). Neutrophil-derived S100A12 is profoundly upregulated in the early stage of acute Kawasaki disease. *The American Journal of Cardiology*.

[82] Ligthart S., Sedaghat S., Ikram M. A., Hofman A., Franco O. H., Dehghan A. (2014). EN-RAGE. *Arteriosclerosis, Thrombosis, and Vascular Biology*.

[83] Burke A. P., Kolodgie F. D., Zieske A. (2004). Morphologic findings of coronary atherosclerotic plaques in Diabetics. *Arteriosclerosis, Thrombosis, and Vascular Biology*.

[84] Yan L., Bjork P., Butuc R. (2013). Beneficial effects of quinoline-3-carboxamide (ABR-215757) on atherosclerotic plaque morphology in S100A12 transgenic ApoE null mice. *Atherosclerosis*.

[85] Katte R., Yu C. (2018). Blocking the interaction between S100A9 protein and RAGE V domain using S100A12 protein. *PLoS One*.

